# The Role of Methylation of Host and/or Human Papillomavirus (HPV) DNA in Management of Cervical Intraepithelial Neoplasia Grade 2 (CIN2) Lesions

**DOI:** 10.3390/ijms24076479

**Published:** 2023-03-30

**Authors:** Andraž Dovnik, Mario Poljak

**Affiliations:** 1 University Clinic for Gynecology and Obstetrics, Maribor University Medical Center, Ljubljanska 5, 2000 Maribor, Slovenia; 2 Institute of Microbiology and Immunology, Faculty of Medicine, University of Ljubljana, Zaloška 4, 1000 Ljubljana, Slovenia; mario.poljak@mf.uni-lj.si

**Keywords:** cervical intraepithelial lesion, human papillomavirus, gene methylation

## Abstract

Cervical intraepithelial neoplasia grade 2 (CIN2) is an intermediate stage between CIN 1, which is a low-grade lesion, and CIN3, which is the immediate precursor of cervical cancer (CC). Traditionally, CIN2 was regarded as a high-grade lesion and was treated with conization or ablative methods. In recent years, there has been a shift in the management of younger patients, who are now more often being managed conservatively due to frequent spontaneous CIN2 regression and possible adverse effects of treatment on future pregnancies. Because the risk of progression to CC still exists with conservative management, a personalized approach is needed to identify patients with a higher probability of progression. In this regard, research has focused on the role of host and human papillomavirus (HPV) gene methylation. This systematic review summarizes the current knowledge regarding conservative CIN2 management focusing on the main methylation markers and its implementation in conservative CIN2 management, and it describes major ongoing longitudinal studies on the subject. The review showed that DNA methylation is an accurate predictor of disease progression and a valid triage tool for HPV-positive women, with CIN2 performing better than triage cytology. Because virtually all CCs are methylation-positive, methylation-negative women at baseline have an extremely low risk of CC.

## 1. Introduction

Cervical intraepithelial neoplasia grade 2 (CIN2) is an intermediate stage between low-risk dysplasia and CIN3, which is considered an immediate precursor of cervical cancer, with a progression rate between 30% and 40% [1]. The classic management of CIN2 is surgical with the excision of the transformation zone, and this approach is highly effective [2]. On the other hand, it is associated with a higher risk of second-trimester pregnancy termination and preterm labor [3]. Due to such complications and the fact that a substantial proportion of CIN2 lesions regress spontaneously, it is important to differentiate between women that would benefit from immediate surgical treatment and those that can forego or substantially postpone invasive treatment. In this regard, extensive research on several triage markers, including methylation markers, has been performed in the past decade [1,3].

We performed a systematic review of the literature on the role of human papillomaviruses (HPV) DNA and/or host gene methylation in the identification of cervical precancerous lesions with a high probability of progression. In addition, we searched for articles dealing with conservative management of CIN2. The literature search was conducted using the MEDLINE electronic database for the search terms “HPV” AND “DNA methylation” AND “cervical precancer”. The literature regarding CIN2 management was searched using the search terms “CIN2” AND “conservative management”. Among the 129 articles identified, we selected 105 articles published up to December 2022. Peer-reviewed articles published in English and containing an abstract were considered, and reference lists were screened for additional relevant citations. We added some additional manuscripts to the reference list due to close association with the content of the manuscript. Full-text versions of all manuscripts were obtained. The systematic review was conducted in accordance with PRISMA guidelines.

The first part of this article summarizes the current knowledge regarding conservative management of CIN2. The second part focuses on the main methylation markers that have been studied to date and the possibilities for implementing methylation markers in conservative management of CIN2. The final part briefly describes ongoing longitudinal studies dealing with the possible role of methylation marker assessment in conservative management of precancerous cervical lesions.

## 2. Cervical Intraepithelial Neoplasia Grade 2 (CIN2) and Its Current Management Strategies

Cervical cancer develops through three grades of cervical premalignant lesions, termed cervical intraepithelial neoplasia 1–3 (CIN1–3) [1]. Treatment of cervical precancerous lesions is performed with the aim of preventing their development into invasive cancer [4]. CIN1 includes low-grade lesions that regress spontaneously within 2 years in more than 60% of cases and are therefore treated conservatively [4,5]. CIN2 and CIN3 are considered high-risk lesions. Although they also have the ability to spontaneous regress, they are usually treated with excisional or ablative procedures [5]. In developed countries, the annual incidence of CIN2/3 is 1.5/1000 women, and the incidence is the highest in the age group between 25 and 29 years old [6]. Although there is consensus that CIN3 is an immediate precursor of cervical cancer, opinions concerning the clinical course, progression rate, and management of CIN2 are highly diverse [3]. By definition, in CIN2, atypical basaloid cells are present in up to half of the epithelial thickness, koilocytes might be present, and mitoses are present in the lower half of the epithelium [7,8]. However, several histological review studies showed that histological diagnosis of CIN2 is non-reliable, much less reproducible, and substantially less valid than CIN3 [9,10,11]. Adjunctive use of immunostaining with p16^INK4a^ (p16), a tumor-suppressor surrogate protein biomarker for high-risk HPV oncogenic activity, to standard hematoxylin and eosin histology significantly improves the accuracy of CIN2 assessment [12,13,14].

Despite all the uncertainties connected with accurate diagnosis of CIN2, both CIN2 and CIN3 are usually treated with excision of the transformation zone, which is highly effective despite the fact that the risk of cervical cancer remains increased up to 20 years after excisional treatment [2]. High-risk lesions can also be treated with ablative techniques with the use of various energy sources. However, this approach is much less commonly used [15].

Excisional treatment increases the risk of pregnancy termination in the second trimester and preterm labor [3]. The age of women having their first child is increasing, and women are often diagnosed with CIN2/3 before their first pregnancy [3]. Therefore, the identification of women that will benefit from excisional treatment is extremely important [3]. Several meta-analyses indicated a higher risk of perinatal complications with excisional treatment of cervical precancerous lesions and early cancer compared to no treatment [16,17,18,19,20,21]. The most recent meta-analysis reported that increased risk of preterm labor is connected with all excisional techniques [22]. Excisional treatment on the cervix is also associated with other short- and long-term complications [23]. For 2–4 weeks after excisional treatment, bleeding occurs in up to 85% of women, and pain occurs in up to 67% [23]. Pain can persist up to 3 months after the procedure. More rarely, patients report vaginal discharge, dyspareunia, and postcoital bleeding [23]. In addition, excisional treatment affects patients’ feelings, with high reported rates of fear about cancer and future fertility [23].

The most recent U.S. guidelines, published in 2020, recommend treatment of all CIN3 lesions [24]. Treatment is also recommended in women with CIN2 lesions, but it can be omitted if the risk of future pregnancy complications exceeds the risk of cancer development [24]. Conservative management of CIN2 lesions is unacceptable in cases where colposcopy of the CIN2 lesion is unsatisfactory and in cases where the lesion extends into the cervical canal. Conservative management of CIN2 lesions is acceptable if the patient is younger than 25. In older patients, conservative management is acceptable in the case of fear of future pregnancy complications. According to the guidelines, conservative management in patients younger than 25 comprises colposcopy and cervical cytology every 6 months [24]. On the other hand, in patients older than 25, colposcopy with HPV testing is recommended. If the CIN2 lesion persists for 2 years or CIN3 is detected during follow-up, treatment is recommended. In cases of regression of CIN2 to CIN1 or less, the follow-up interval can be prolonged to 1 year. When treatment is necessary, excisional treatment is recommended, but ablative techniques are also acceptable [24]. According to the most recent World Health Organization guidelines, ablative treatment is unacceptable if the lesion is present in more than 75% of the transformation zone surface and in cases where the lesion extends to the cervical canal [25]. The rate of disease recurrence is 26.6% after excisional treatment and 31.0% after cryotherapy [26]. A Cochrane meta-analysis did not reveal statistically significant differences in recurrence rates among various techniques. However, excisional treatment provides an important benefit: a reliable sample for histological assessment [27].

CIN2 has a relatively high rate of spontaneous regression [3,24]. The regression rates of CIN2 reported by various research groups are presented in Table 1.

The largest meta-analysis studied 3160 patients from 36 studies, of which there were seven randomized studies, 16 prospective cohorts, and 13 retrospective cohorts [3]. After 24 months of observation, 50% of CIN2 lesions spontaneously regressed, 32% persisted, and 18% progressed to CIN3+ (CIN3 or invasive cervical cancer). Twenty-nine studies defined progression as histological diagnosis of CIN3 or worse, and seven studies defined progression as high-risk cytological changes [3]. Regression was defined as normal histological or cytological findings at the end of follow-up in 25 studies, CIN1, or cytological finding of low-grade squamous intraepithelial lesion (LSIL) or atypical squamous cells of undetermined significance (ASC-US) in 17 studies, and 6 studies included both definitions of regression. The majority of regressions occurred within the first year of follow-up. Regression rates were particularly high in patients younger than 30, for whom they reached 60%. In the case of progression, the great majority of lesions progressed to CIN3, whereas only 15 patients (0.5%) developed invasive cervical cancer by the end of the follow-up. Among these 15 patients, there were 13 with the FIGO IA1 stage, and only 2 patients (0.06% of the total cohort) presented with more advanced disease. The authors concluded that conservative management of CIN2 is feasible and safe in younger women [3].

After the publication of a meta-analysis in 2018 [3], a few additional retrospective and prospective studies also showed the feasibility of conservative management of CIN2 lesions [28,29,30,31,32,33,36]. A U.S. retrospective analysis reported on 2417 women between 21 and 39 years old with CIN1/2, CIN2, and CIN2/3 that were followed every 6 months with colposcopy and cytology [36]. Regression occurred in 50% of cases. These women were retained in the follow-up due to CIN1 lesions or persistent positive HPV. Thirty percent of the patients were treated due to lesion persistence or progression. Only six women developed invasive cancer, and half of these patients were non-responders [36]. A Danish retrospective analysis included 6721 women with CIN2 in the period between 2008 and 2011, and 6399 women with CIN2 in the period between 2014 and 2017 between 18 and 44 years old [28]. The percentage of women managed conservatively increased from 29.6% to 53.6%. In the period between 2008 and 2011, 41.8% of lesions regressed, 40.9% of lesions persisted, and 16.6% of lesions progressed. In the period between 2014 and 2017, spontaneous regression occurred in 46.7% of lesions, 35.5% persisted, and 17.1% progressed. The rate of regression was similar in age groups above and below 30 years old [28]. Another U.S. retrospective analysis of 154 women with CIN2 and CIN3 younger than 24 showed spontaneous regression in 74.7% of cases with CIN2, with the average time of regression of 10.8 months [32]. Korean authors reported regression rates in 75 women with CIN2 and 140 women with CIN3 diagnosed during pregnancy [34]. Women were followed during pregnancy and postpartum. After pregnancy, CIN3 was diagnosed in 89 patients and invasive cancer in three patients. CIN2 was diagnosed in 15 patients and CIN1 in 10 patients. An important risk factor for persistence of CIN2+ was persistent infection with high-risk HPV [34]. A Dutch study of 56 patients diagnosed with CIN2 between 2000 and 2013 showed a 61% rate of spontaneous regression [29]. The rate of spontaneous regression was higher in nulliparous and non-smoking women [29]. A Spanish prospective observational study of 214 patients with histologically confirmed CIN2 followed for 2 years showed a regression rate of 73.5%, whereas 11.7% progressed to CIN3 [35].

## 3. HPV-Mediated Cervical Carcinogenesis

In the great majority of cases, the development of cervical cancer is a long-term process that follows persistent infection of the uterine cervix with high-risk HPV types [37,38]. HPV are double-stranded DNA viruses, and the viral DNA codes two groups of proteins: early (E1–7) and late (L1 and L2) proteins [37,39]. Early proteins E6 and E7 interact with host tumor suppressor genes involved in mechanisms of cellular proliferation, and proteins E1 and E2 mediate viral replication [37,40]. Late proteins L1 and L2 form viral capsids [39,40].

HPV can cause productive or transforming infections [41]. Productive infection mostly causes CIN1 and a small subset of CIN2, and most of these lesions undergo spontaneous regression within 1 to 2 years. On the other hand, transforming lesions represent the remainder of CIN2 and CIN3 lesions. They can be divided into early and advanced transforming lesions depending on their short-term progression risk [41]. In contrast to early transforming lesions, advanced transforming lesions are characterized by high methylation levels, more than 5 years of preceding HPV infection, and a lack of E4 expression [1,41,42].

HPVs have a tropism for squamous epithelium and initially infect basal cells of the transformation zone of the cervix [39,43]. The virus is internalized by endocytosis, and it then enters the nucleus through defects in the nuclear membrane [39]. The effect of oncogenic E6 and E7 causes deregulation of the cell cycle by interacting with tumor suppressor genes and hence causes uncontrolled cell progression to the suprabasal layers [44,45]. As the proliferating cells progress to higher layers of the epithelium, the virus expresses E1 and E2 genes, which support productive amplification of the viral genome [44]. The expression of the L1 and L2 proteins in the upper layer of the epithelium helps assemble viral particles, which are then ready for further transmission [44].

The most important early event in cervical carcinogenesis is the integration of HPV DNA into the host genome [45]. Integration most frequently affects E1 and E2 genes, which are physically disrupted. Because E2 has the function of negatively controlling the expression of oncogenic E6 and E7 proteins, inactivation of E2 causes increased expression of E6 and E7 [46]. E6 and E7 cause neoplastic transformation through various pathways, and as such they are a driving force in cervical carcinogenesis [47]. E6 targets p53, which is the most important human tumor suppressor gene and is also involved in the process of apoptosis. The degradation of p53 occurs through ubiquitination with E6-associated protein (E6AP). This leads to the evasion of preventive cell cycle check points by the inhibition cyclin-dependent kinase (CDK) inhibitors p27 and p21 and causes the cells to divide uncontrollably [47]. E7 targets tumor suppressor retinoblastoma protein pRb, which is normally bound to the transcription factor E2F [37]. E2F transcribes S-phase proteins such as cyclin A, cyclin E, CDK 4/6 inhibitor, and p16. The action of E7 leads to uncontrolled cell entry into S-phase and therefore uncontrolled cell division [47]. E6 and E7 proteins are also implicated in the evasion of apoptosis. They were found to influence the MAPK and mTOR signaling pathways and cause deregulation of proliferation signaling pathways. In addition, they are also involved in the processes of tumor neoangiogenesis and the epithelial-to-mesenchymal transition (EMT) [47].

## 4. HPV-Mediated DNA Methylation

A persistent infection with high-risk HPV types causes several epigenetic changes in both host and HPV DNA [48,49]. An epigenetic change is defined as a change in the expression of host or viral genes without changes of the encoding DNA sequence [37]. DNA methylation is the most extensively studied epigenetic change in HPV-related cancers [50]. During DNA methylation, DNA methyltransferase enzymes covalently add methyl groups to cytosine preceding guanine (CpG) [51]. The regions that are rich in CpG are usually located in protein-coding gene promoters [51]. The methylation of host tumor suppressor genes gradually increases during cervical pathogenesis [1]. Five methylation targets that have been shown to be important in methylation-associated gene silencing during cervical carcinogenesis include FAM19A4, miR124, CADM1, MAL, and PAX1 [1,52].

Here we briefly review the available literature regarding the performance of methylation analysis of various host tumor suppressor genes and HPV genes in the detection of cervical precancerous lesions and invasive cancer with the focus on clinically validated methods/protocols/assays. An overview of the most promising host and HPV DNA methylation assays with a brief summary of the main findings and selected references is presented in Table 2.

### 4.1. MicroRNA

MicroRNA (miRNA) is a short non-coding RNA that is involved in the expression of protein coding genes by base pairing with target mRNA at its 3′ untranslated region (UTR) [71]. HPV-encoded genes influence the expression of host cell miRNA. In addition, miRNA is associated with HPV insertion sites [71]. The rate of miRNA has-miR-124 methylation, which is associated with decreased expression of this miRNA, ranged from 0% in normal cervical tissue to 58.5% in CIN3 and more than 90% in cervical cancer in a study of 139 cervical tissue specimens [71]. The expression of various other types of host and HPV miRNA in plasma and cervical samples has been studied by other research groups. The authors reported conflicting results with regard to the association between miRNA expression and the grade of the lesion [72,73,74,75,76,77,78,79,80,81].

### 4.2. FAM19A4/miR124-2

FAM19A4 is a host gene that encodes a small protein associated with inflammation and stress. The methylation of this gene has been extensively evaluated in HPV screen-positive women as a triage method [52,82,83]. In a Dutch study, the methylation of FAM19A4 was compared among groups of patients with cervical cancer, early and advanced high-grade squamous intraepithelial lesion (HSIL), depending on the duration of preceding HPV infection [84]. All cases of advanced HSIL and cervical carcinomas were methylation-positive, whereas methylation positivity was less than 50% in early HSIL [84]. In a Chinese cohort study, the methylation of FAM19A4 was compared among 66 women without cervical (pre)cancerous lesions, 31 women with low-grade squamous intraepithelial lesion (LSIL), and 57 women with HSIL [52]. The methylation scores were significantly higher in HSIL lesions compared to the cases without cervical lesions (56.2% vs. 10.6%, *p* < 0.05) [52].

The methylation of FAM19A4 and mir-124-2 for the detection of CIN3+ has also been studied in combination and has shown high sensitivity and specificity in self-collected and clinician-collected specimens [53,85]. A negative combined FAM19A4/mir-124-2 methylation test was associated with a low long-term risk of cervical cancer in a Dutch longitudinal study [53]. Another recent Dutch population-based study compared methylation of FAM19A4/mir124-2 to cytology as a triage method for the detection of CIN3+ [54]. The sensitivity of methylation was comparable to cytology, but the specificity was lower. The combination of these two tests achieved higher sensitivity but was associated with higher colposcopy referral rates. A negative methylation test was associated with lower long-term cervical cancer risk compared to negative cytology [54]. A recent European retrospective study of 371 HPV-positive women with CIN2+ evaluated the diagnostic utility of the FAM19A4/mir-124-2 methylation marker in distinguishing lesions in need of treatment [55]. The authors reported a high specificity of FAM19A4/mir-124-2 in women under 30 in detecting non-productive CIN2/3 lesions [55]. The same researchers also evaluated the association of p16 and Ki67 immunoscore with FAM19A4/mir-124-2 methylation status and immunohistochemical HPV E4 expression on 497 women with histologically confirmed high-grade cervical lesions [86]. They reported increased methylation positivity from CIN2 to CIN3 (63.0% vs. 79.1%, *p* < 0.001) [86]. Considering the accumulated evidence from key studies, FAM19A4/mir-124.2 methylation may be used as a valid triage method for HPV screen-positive women because it provides a very high negative predictive value for the development of cervical cancer. In addition, it may be particularly useful in distinguishing younger women with high-risk lesions that do not need immediate surgical treatment.

### 4.3. CADM1/MAL

CADM1 is a tumor-suppressor gene involved in cell-to-cell adhesions and is a member of the immunoglobulin superfamily [87,88]. The frequency and density of CADM1 promoter methylation increases with high-grade precancerous lesions and cancer compared to normal tissue, and it ranges from 5% in normal tissue to more than 80% in cervical cancer lesions [88,89]. Higher density and frequency of CADM1 promoter methylation is associated with decreased gene expression [88].

Another tumor suppressor gene with reduced expression during cervical carcinogenesis is T-lymphocyte maturation-associated protein (MAL), which is associated with apical transport of membrane proteins [87,90]. MAL promoter methylation was found in 9% of low-grade lesions, about half of CIN3, and 90% of squamous cell cancers in a study of 274 cervical biopsy specimens of various grades [90].

The methylation status of CADM1 has also been studied in combination with MAL [56,57,58,91]. In a Dutch study including 221 women, among whom 167 had CIN1 or normal histology, 54 had CIN2/3 and 44 had cervical cancer; higher methylation levels of CADM1 and MAL were found in CIN2/3 lesions and cervical cancer samples in comparison to ≤CIN1 (5.3- and 6.2-fold increased methylation level of CADM1 and MAL; *p* < 0.0005). In addition, methylation levels in patients with high-risk precancerous lesions were higher in cases with more than 5 years of preceding HPV infection, which is considered a surrogate marker for the age of the lesion (3.0- and 11.5-fold increased methylation level for CADM1; *p* = 0.023; 3.6- and 13.6-fold increased methylation level for MAL; *p* = 0.005) [56]. This study showed that methylation levels increase with the duration of preceding HPV infection and with lesion severity, and that methylation analysis can help separate early lesions from more advanced high-grade lesions [56]. Another Dutch study compared the performance of CADM1/MAL methylation and cytology as a triage test for the detection of high-grade lesions in HPV-positive women. The performance of cytology and methylation testing was comparable. Higher sensitivity was achieved when cytology and methylation were used in combination [58]. In addition, this combination was analyzed in a cohort of women with more than one cervical biopsy [57]. Because a single woman can experience infection with different HPV types, multiple cervical lesions of various grades can be present. This study showed that positive CADM1/MAL methylation status was present in CIN2/3 lesions and not in normal biopsy on the same cervix in almost 90% of patients with multiple biopsies [57]. The methylation analysis performed in a cervical scrape was strongly representative of the most advanced lesion on a cervix, particularly cervical cancer and CIN3 [57]. Moreover, this combination of host genes with the addition of death-associated protein kinase 1 (DAPK1), which is a positive modulator of apoptosis, was also studied in Brazil. Methylation levels correlated with the severity of lesions [92]. The methylation levels of CADM1/MAL thus correspond to the severity of the lesion and also to the duration of the preceding HPV infection. This panel can also be used as a triage method for HPV screen-positive women.

Furthermore, several researchers studied the combined methylation status of CADM1, MAL, and miR124 [93,94,95,96]. The methylation rates of all genes were higher in more advanced lesions in all studies [93,94,95]. A prospective study including women with normal biopsy, various grades of cervical precancerous lesions, and cases of cervical cancer reported that the positivity rates for all three genes were similarly high in CIN3 and cervical cancer cases and similarly low in low-grade lesions and normal histology samples (51.6% and 57.4% vs. 10.5% and 0.0% for CADM1; *p* < 0.001; 60.0% and 92.8% vs. 10.5% and 0.0% for MAL; *p* < 0.001; 68.3% and 78.5% vs. 15.7% and 12.5% for miR124; *p* < 0.001) [93]. The methylation pattern of CIN2 lesions was similar to CIN3 and cervical cancer for miR124 and MAL, but it resembled the methylation pattern of low-grade lesions for CADM1 [93].

### 4.4. Other Host Cell DNA Methylation Markers

The methylation levels of the POU Class 4 Homeobox 3 (POU4F3) gene are increased in cervical cancer, which may indicate a tumor-suppressor role of this gene despite the fact that its function in carcinogenesis is unknown [97]. The methylation of POU4F3 was evaluated in a Hungarian study of 5384 liquid-based cytology samples. The authors reported a sensitivity for the detection of CIN2+ of 88.2% and a specificity of 72.9% [59].

A Taiwanese cohort study of 73 patients evaluated the methylation levels of paired box gene 1 (PAX1) in cervical precancer and invasive cancer and compared it to high-risk HPV results generated using the digene Hybrid Capture 2 HPV DNA Test (HC2; Qiagen, Gaithersburg, MD, USA). The methylation of PAX1 provided a higher specificity for detecting cervical cancer compared to HC2 [60]. These results were confirmed in another recent Taiwanese study [61]. PAX1 and cytology had similar specificity (more than 92%) as triage tools for the detection of CIN3+ in HPV-positive women. The specificity of PAX1 was higher than that of partial HPV16/18 genotyping (92.5% vs. 75.8%, *p* ˂ 0.05). A Chinese study of 462 cases of ASC-US cytology revealed higher sensitivity and specificity values of PAX1 methylation in the detection of CIN2+ compared to HC2 [62].

A methylation panel consisting of ASTN1, DLX1, ITGA4, RXFP3, SOX17, and ZNF671 was also evaluated [98,99]. In a small German study of 79 patients, the sensitivity of this six-member panel for the detection of CIN3+ was 64.8%, and the specificity was 94.6% [99].

### 4.5. High-Risk HPV DNA Methylation Markers

Less than 5% of incident HPV infections progress to CIN3, and the methylation of some of the HPV genes is an important biomarker of this subset of clinically relevant transforming HPV infections [63]. The available literature is consistent with regard to the increased levels of HPV16L1 methylation in cervical cancer compared to low-grade lesions [100,101]. Torres-Rojas et al. reported a 33.0% HPV16L1 methylation rate for low-grade lesions and 58.6% in cervical cancer (*p* < 0.0001) [101]. In addition, increased methylation levels of E2, L1, and L2 of HPV18, HPV31, and HPV45 were found in CIN3 lesions in comparison to CIN2 or less in a U.S. case-control study of 92 women with CIN3 compared to 96 women with CIN2 or less [64]. A British study compared methylation levels of 528 patients within a colposcopy referral population that tested positive for HPV18, HPV31, and HPV33 [65]. Within this cohort, 249 patients had CIN2+. Significantly higher levels of L1 and L2 methylation for all three HPV types were present in CIN2+ [65]. The methylation of HPV18 and HPV31 was lower in cases of multiple infections compared to a single infection [65]. The methylation levels of CpG sites within the HPV52 genome were analyzed in a U.S. case-control study of 50 cases of CIN3 and 39 cases without high-grade disease [63]. The most significant difference in methylation levels was found in HPV52L1 [63]. Another U.S. case-control study compared the methylation levels of 12 HPV types [66]. The patients were selected from a larger cohort with the aim of comparing methylation levels in L1 and L2 of HPV types 16/18/31/33/35/39/45/51/52/56/58/59 between patients with CIN3 or adenocarcinoma in situ (AIS) and normal controls. For each of the 12 HPV types, 30 cases of CIN3/AIS and 30 normal controls were selected. Within L1 and L2 of 12 carcinogenic HPV types, next-generation bisulfite sequencing was performed on CpG sites. Methylation levels were significantly higher in CIN3/AIS compared to normal controls in all HPV types [66]. AUCs were calculated for the top sites and ranged from 0.71 (HPV51 and HPV56) to 0.86 (HPV18) [66]. This study showed an association between CIN3/AIS and elevated methylation levels of L1 and L2 genes for HPV16, HPV18, HPV31, HPV33, and HPV45 [66].

### 4.6. The Combination of Host and HPV Methylation Markers

A combination of host and HPV gene methylation using the S5 classifier (host gene EPB41L3 and genes of HPV16, HPV18, HPV31, and HPV33) for the detection of high-grade cervical precancerous lesions has been studied in a colposcopy referral population and in population-based screening studies [67,68,69,70]. The S5 classifier had higher sensitivity and comparable specificity for the detection of CIN2+ compared to HPV16/18 genotyping [67]. In addition, the ability of the S5 classifier to reduce colposcopy referrals by 50% with the potential to increase cost-effectiveness was shown [69].

A recent meta-analysis of 43 studies included more than 16,000 women [102]. Nine studies evaluated DNA methylation markers among women with HPV16 infection, seven studies included women with abnormal cytological results, and twenty studies included women with positive HPV DNA results. Among the host genes analyzed were CADM1, MAL, EPB41L3 alone or as part of the S5 classifier, PAX1, SOX1 (sex determining region Y, box 1), FAM19A4, and POU4F3. Ten of the studies included reported the association of HPV16L1 and/or L2 with CIN2+ and CIN3+ [102]. Women with CIN2 and CIN3 were more likely to be methylation-positive compared to ≤CIN1 (OR = 2.83 and 7.92, respectively). The comparison between groups with CIN2 and CIN3 showed a higher risk of positive methylation among women with CIN3 (OR = 2.95). The pooled sensitivity and specificity of DNA methylation markers were 62.2% and 75.9% for CIN2+, and 70.5% and 74.7% for CIN3+, respectively [102].

## 5. The Role of Methylation Markers in Conservative Management of Women with CIN2

It is still not known whether CIN2 lesions with high methylation levels are more aggressive compared to histologically similar lesions with low methylation levels. Longitudinal studies are needed to further define the possible role of methylation panels in the conservative management of CIN2 lesions [87]. A Finnish longitudinal study evaluated the utility of the S5 classifier methylation panel on 149 women with CIN2 [103]. In this study, follow-up visits were scheduled every 6 months for 2 years. It found that 16.8% of lesions progressed to CIN3+, 24.2% persisted as CIN1/2, and 59% regressed to less than CIN1. The study revealed that the S5 classifier performed better than HPV16/18/31/33 genotyping in predicting progression versus regression of CIN 2 lesions [103].

A recently published Dutch prospective observational study included 114 women between 20 and 53 years old with CIN2/3 that were prospectively followed for 24 months [104]. Clinical regression was defined as the absence of CIN2+ at the end of the follow-up or a negative HPV with normal cytology if no histology was available. A negative FAM19A4/miR124-2 methylation result at the start of the study was associated with a higher degree of clinical regression compared to a positive result (74.7% vs. 51.4%; *p* = 0.013) [104]. In the group of women with a negative methylation test, the regression rate was the highest in the group with ASCUS/LSIL cytology (88.4%) and negative HPV16 (85.1%). This study showed that methylation-negative cases of CIN2+ are more likely to regress than methylation-positive cases. Based on these results, DNA methylation could be used as a triage for HPV-positive women with ASCUS/LSIL cytology [104].

Our research group has started a similar study in young women ≤35 years old with histologically confirmed CIN2. The lesion on the uterine cervix must be completely visible and cover less than 75% of the transformation zone. Concomitant histologic or cytologic glandular changes represent an exclusion criterion. HPV DNA testing with HC2, full HPV genotyping by Anyplex II HPV28 Detection Kit (Seegene, Seoul, South Korea), and methylation status analysis of FAM19A4 and miR124-2 using the QIAsure Methylation Test (Qiagen) are performed at enrollment. The primary endpoint of our study is the regression rate with regression defined as ≤CIN1 after 2 years of follow-up. The secondary endpoint is the association between HPV type(s) and methylation status and the probability of regression.

## 6. Conclusions

With the high rate of spontaneous regression of CIN2 lesions, new biomarkers are needed to further individualize their management. The British Society for Colposcopy and Cervical Pathology recently conducted a survey among its members regarding the management of CIN2 lesions [105]. Two-thirds of clinicians offered their patients with CIN2 conservative management despite the lack of formal guidelines supporting such practice. The majority of survey participants agreed that women’s age over 40, large size of the lesion, and the presence of HPV16 or HPV18 are contraindications for conservative CIN2 management.

Ongoing and planned large longitudinal studies from different parts of the world including a variety of methylation markers should soon provide further insights for the informed management of women with CIN2 lesions, safely differentiating between women that would benefit from immediate surgical treatment and those that can opt for conservative CIN2 management.

## Figures and Tables

**Table 1:** Overview of studies evaluating the rate of spontaneous regression of CIN2 lesions.

Study	Number of Women	Type of Study	Follow-Up Duration (Months)	Regression Rate (%)	Persistence Rate (%)	Progression Rate (%)	Reference
Tainio et al.	3160	Meta-analysis	24	50	32	18	[3]
Skorstengaard et al. (2008–11)	1989	Retrospective study	10	41.8	40.9	16.6	[28]
Skorstengaard et al. (2014–17)	3427	Retrospective study	10	46.7	35.5	17.1	[28]
Koeneman et al.	56	Retrospective study	24	61	NA	NA	[29]
Loopik et al.	401	Retrospective study	16–33	73.1	12.7	14.2	[30]
Godfrey et al.	100	Retrospective study	22	57	30	13	[31]
Lee et al.	99	Retrospective study	24	74.4	NA	NA	[32]
Tjandraprawira et al.	175	Retrospective study	22.6	77.3	13.4	9.3	[33]
Kyung Hong et al.	47	Retrospective study	Follow-up during pregnancy	44.7	19.1	36.2	[34]
Salvado et al.	291	Retrospective study	24	73.5	14.8	11.7	[35]

Abbreviations: CIN = cervical intraepithelial neoplasia; NA = data not available.

**Table 2:** Overview of the most promising host and HPV DNA methylation assays with a brief summary of the main findings and key references.

DNA Methylation Assay	Studies	Comment
FAM19A4/miR124-2	[53,54,55]	A negative test result associated with lower long-term cervical cancer risk compared to negative cytology; high NPV for the development of cervical cancer.
CADM1/MAL	[56,57,58]	Methylation levels correspond to the severity of the lesion and to the duration of pre-existing HPV infection.
POU4F3	[59]	High sensitivity and specificity for the detection of CIN2+.
PAX1	[60,61,62]	Used in Asian populations only; higher specificity compared to HPV genotyping for the detection of CIN3+.
HPV DNA	[63,64,65,66]	Higher sensitivity and comparable specificity for the detection of CIN2+ compared to HPV16/18 partial genotyping. Positive association between CIN3/AIS and elevated methylation levels of L1 and L2 for HPV16, HPV18, HPV31, HPV33, and HPV45.
S5 classifier (host gene EPB41L3 and genes of HPV16, HPV18, HPV31, and HPV33)	[67,68,69,70]	Higher sensitivity and comparable specificity for the detection of CIN2+ compared to HPV16/18 partial genotyping.

Abbreviations: HPV = human papillomaviruses; NPV = negative predictive value; CIN = cervical intraepithelial neoplasia; AIS = adenocarcinoma in situ.
